# Timely and individualized heart failure management: need for implementation into the new guidelines

**DOI:** 10.1007/s00392-021-01867-2

**Published:** 2021-05-13

**Authors:** Amr Abdin, Johann Bauersachs, Norbert Frey, Ingrid Kindermann, Andreas Link, Nikolaus Marx, Mitja Lainscak, Jonathan Slawik, Christian Werner, Jan Wintrich, Michael Böhm

**Affiliations:** 1 Klinik Für Innere Medizin III-Kardiologie, Angiologie Und Internistische Intensivmedizin, Universitätsklinikum Des Saarlandes, Kirrberger Strasse 100, 66421 Homburg, Germany; 2 Department of Cardiology and Angiology, Hannover Medical School, Hannover, Germany; 3 Department of Internal Medicine III, University of Heidelberg, 69120 Heidelberg, Germany; 4 Department of Internal Medicine I, Cardiology, University Hospital Aachen, RWTH Aachen University, Aachen, Germany; 5 Division of Cardiology, General Hospital Murska Sobota, Murska Sobota, Slovenia

**Keywords:** Heart failure, Outcomes, Treatment, Management

## Abstract

Due to remarkable improvements in heart failure (HF) management over the last 30 years, a significant reduction in mortality and hospitalization rates in HF patients with reduced ejection fraction (HFrEF) has been observed. Currently, the optimization of guideline-directed chronic HF therapy remains the mainstay to further improve outcomes for patients with HFrEF to reduce mortality and HF hospitalization. This includes established device therapies, such as implantable defibrillators and cardiac resynchronization therapies, which improved patients' symptoms and prognosis. Over the last 10 years, new HF drugs have merged targeting various pathways, such as those that simultaneously suppress the renin–angiotensin–aldosterone system and the breakdown of endogenous natriuretic peptides (e.g., sacubitril/valsartan), and those that inhibit the *I*_f_ channel and, thus, reduce heart rate (e.g., ivabradine). Furthermore, the treatment of patient comorbidities (e.g., iron deficiency) has shown to improve functional capacity and to reduce hospitalization rates, when added to standard therapy. More recently, other potential treatment mechanisms have been explored, such as the sodium/glucose co-transporter inhibitors, the guanylate cyclase stimulators and the cardiac myosin activators. In this review, we summarize the novel developments in HFrEF pharmacological and device therapy and discuss their implementation strategies into practice to further improve outcomes.

## Novel heart failure treatments

### Sodium/glucose cotransporter 2 inhibitors

Sodium/glucose cotransporter 2 (SGLT2) inhibitors reduce cardiovascular (CV) mortality and heart failure (HF) hospitalizations in patients with diabetes mellitus (DM) in studies were signs and symptoms of HF were not required for inclusion [1, 2]. These findings led to the hypothesis that SGLT2 inhibitors may represent an effective treatment of HF independent of the DM status [3]. Accordingly, prospective, controlled trials were designed to investigate the effect of SGLT2 inhibitors, dapagliflozin (DAPA-HF, 3) and empagliflozin (EMPEROR-Reduced, 4) in HF with reduced ejection fraction (HFrEF) patients. Dapagliflozin resulted in a 26% reduction in the primary endpoint of CV death, HF hospitalizations and urgent presentations for worsening HF [3]. This resulted in a "number needed to treat" (NNT) of 21 patients, i.e., 21 patients need to be treated to prevent one of these events. In the DAPA-HF trial, all components of the primary endpoint were similarly reduced and there was no heterogeneity between subgroups [3]. The treatment effects of dapagliflozin were not different across all groups of renal function at baseline [5]. Recently, the EMPEROR-Reduced trial showed a consistent 25% reduction in the primary endpoint of CV death and HF hospitalizations when empagliflozin was added to established therapy in symptomatic patients with HFrEF [4]. In EMPEROR-Reduced, there was a 30% reduction in all HF hospitalizations (the first and all subsequent) and a significant reduction in worsening of renal function over time, reflecting a nephro-protective effect. Similarly, endpoints consisting of dialysis requirement, decline in estimated glomerular filtration rate (eGFR) by >  = 40% or death from renal or CV disease were reduced by 50% [4, 6]. Since phase II trials have shown that a natriuretic and glucosuric effect occurs not only in DM but also in non-DM patients, DAPA-HF and EMPEROR-Reduced were conducted in patients with and without DM [3–6]. Two large analyses showed that effects on endpoints for both drugs [7, 8] were not different in patients with or without DM. There was also no difference in the treatment effect of empagliflozin or dapagliflozin across the spectrum of HbA1C levels [7, 8]. Additionally, the marked reduction of CV death and HF hospitalizations and the renal end point (slope of the change in eGFR) was similar in patients with and without ARNI treatment at baseline [9]. Furthermore, in HF, low blood pressure is a problem that often discourages physicians from prescribing guideline-guided therapies. This is related to a fear of adverse drug effects as most of effective HF agents and to the fact that low blood pressure associates with poor prognosis [10–12]. Since SGLT2 inhibitors also lower blood pressure in hypertensive patients [13], there was concern that SGLT2 inhibitors also lower blood pressure in HF to a range that leads to intolerance [14]. However, DAPA-HF trial showed only a small drop (1–2 mmHg) in blood pressure, which was negligible in patients with low baseline blood pressure (< 110 mmHg). At follow-up, blood pressure values rose again [15]. Correspondingly, there were no significant differences in side effects in the individual blood pressure groups and no increased discontinuation rates due to low blood pressure [15]. Taking together, this therapy appears to be cost-effective [16], and so far, no subgroup showed more or less therapeutic effects in both trials. For the SGLT1/2 inhibitor, sotagliflozin, the SCORED trial including patients with DM with and without HF [17] and SOLOIST (HF trial with inclusion of patients immediately after decompensation in patients with DM) [18] were conducted. The endpoint of reduction of CV death and HF hospitalizations was also shown. These trials were stopped early due to the COVID-19 pandemic and loss of sponsoring and recruited less than half of the planned number of patients. Nevertheless, the results were striking enough to confirm the data from the DAPA-HF [3] and EMPEROR-Reduced trials [4].

### Vericiguat (guanylate cyclase activator)

In CHF, the impaired formation of cyclic guanosine monophosphate (cGMP), contributes to endothelial dysfunction and possibly causes myocardial relaxation impairment [19]. Vericiguat is an orally available guanylate cyclase stimulator that activates the formation of cGMP via direct stimulation of guanylate cyclase [19]. As such, it directly stimulates the formation of nitric oxide (NO), but also increases the sensitivity to endogenous NO [19]. Preliminary studies have shown that vericiguat is safe and leads to a decrease in N-terminal pro B-type natriuretic peptide (NT-proBNP) concentrations [20]. In light of these encouraging results, the VICTORIA trial was designed to include patients with more severe HF, some of whom were randomized immediately after acute decompensation [21]. The VICTORIA trial showed a significant 10% reduction in the combined endpoint of CV death and HF hospitalizations, although the individual endpoint components were not significantly reduced [21]. However, given the high incidence of events in the placebo arm, which was up to 50% over 20 months, the absolute risk reduction was about 4%, which is in line with the absolute risk reduction observed in other trials. Whilst the discussion about the position of vericiguat amongst HFrEF therapy is ongoing, it is possible that this novel drug may have a role in the treatment of severe HFrEF patients in the aftermath of a recent hospitalization added to other established HFrEF therapies. Vericiguat has been approved by the Food and Drug Administration (FDA), while approval by European Medicines Agency (EMA) is pending.

### Omecamtiv mecarbil (myosin activator)

Omecamtiv mecarbil activates the interaction of myosin with actin, leading to an increase in contractility force and contraction duration [22]. Shortening of systolic ejection time is a described phenomenon in HF [23] and led to the speculation that beneficial effects in HF may be achieved via directly modulating ejection time. The Chronic Oral Study of Myosin Activation to Increase Contractility in Heart Failure (COSMIC-HF) [23] was a phase II trial that showed a decrease in NT-pro-BNP levels, heart rate and in ventricular dimensions [23]. More recently, the Cardiac Myosin Activation with Omecamtiv Mecarbil in Systolic Heart Failure (GALACTIC-HF) trial studied 8256 patients in a randomized fashion to omecamtiv mecarbil or placebo [24]. There was a nominal (8%) reduction in the composite endpoint of CV death and HF hospitalization, and this effect was formally significant (*p* = 0.03). The components of the primary endpoint, however, and the all-cause mortality were not significantly reduced. GALACTIC-HF showed that this medication is safe, and despite the inclusion of patients with baseline blood pressure of 80 mmHg, there was no drop in blood pressure with omecamtiv mecarbil. Furthermore, GALACTIC-HF included patients who were acutely decompensated, with 30% of patients randomized while still in hospital [25]. Unpublished subgroup analyses indicate that patients in sinus rhythm with low (ejection fraction) EF below the median of 28% and with particularly elevated NT-pro-BNP may benefit from this therapy [25]. Although the study is formally positive, an approval by drug authorities and further clinical development is unknown at this time.

### Intravenous iron therapy

Iron deficiency is present in approximately 50% of patients with HF and is associated with reduced exercise capacity and quality of life independently of anemia [26–28]. Previous studies showed beneficial effects of intravenous iron in the preparation form of intravenous ferri-carboxymaltose on exercise capacity and quality of life and in non-statistically preplanned analyses with trends of reduction in hospitalization and death [28]. The recently published AFFIRM-AHF study showed that among patients with acute HF and iron deficiency, intravenous ferric carboxymaltose was associated with a numerical reduction in total HF hospitalizations and CV death. The trial narrowly missed its primary endpoint of total hospitalizations and CV death but did demonstrate a significant 26% drop in total HF hospitalizations [29]. Interestingly, recruitment was also affected by the COVID-19 pandemic. The study was stopped in the countries as soon as the first COVID-19 case occurred there. Nevertheless, the authors conclude that with the concerted approach to the COVID-19 epidemic by the European Society of Cardiology (ESC) [30], the results show a reliable decrease in the hospitalization rate.

## Sudden cardiac death in HF patients: risk stratification and device therapy

Implantable cardioverter-defibrillator (ICD) Implantation is the main therapy for prevention of sudden cardiac death (SCD) in many patients with HF and left ventricular ejection fraction (LVEF) < 35% [31, 32]. In patients with HFrEF, improvements in pharmacological therapies might lead to an improvement in LVEF and consequently, some patients lose their ICD indication for primary prevention of SCD.

Moreover, the DANISH trial showed that ICD for primary prevention did not show significant beneficial effects on CV and all-cause death in very well-treated, including cardiac resynchronization (CRT), patients with non-ischemic dilated cardiomyopathy [31], whereas only the incidence of SCD was significantly reduced (*p* = 0.005). However, an exploratory subgroup analysis showed a significant survival benefit for patients ≤ 70 years (*p* = 0.03). Interestingly, a recently published multicenter European study emphasizes the importance of scarring for assessing the risk of SCD beside LVEF in HF patients [33]. Patients with a CRT indication were prospectively included in this observational study. Cardiac magnetic resonance imaging (CMR) was performed in all patients. The primary endpoint was a composite of sustained ventricular tachycardia (VT) / ventricular fibrillation (VF), adequate ICD therapy, or SCD. A total of 218 patients with LVEF of 26 ± 7% were analyzed with a median follow-up of 45 months. A myocardial scar was detected in 95% in patients with ischemic cardiomyopathy and 45% in patients with non-ischemic cardiomyopathy. Scar was detectable in 83% of patients who met the primary endpoint. Additionally, the scar detectable on MRI was the only significant predictor for the primary endpoint regardless of LVEF response to CRT (odds ratio, 27.7; 95% CI 3.8–202.7). Consistent with previous reports, this study underlines the importance of a myocardial scar in the occurrence of VT/VF in patients with reduced LVEF [33]. Therefore, scar assessment should be considered as an important parameter for SCD risk stratification in addition to LVEF.

Furthermore, it is still currently unclear which patients with a CRT indication actually benefit from a defibrillator in addition to the pacemaker. A recent study analyzed 45,679 patients treated with a CRT-P or CRT-D system for primary prevention in France between 2010 and 2017 [34]. During a follow-up of 913 days, the incidence of mortality was higher in CRT-P (12%) than in CRT-D patients (7%). Nonetheless, there was no significant mortality difference between CRT-P and CRT-D in patients older than 75 years with non-ischemic cardiomyopathy. In contrast, CRT-P patients younger than 75 years with non-ischemic cardiomyopathy showed higher mortality than CRT-D patients (*p* = 0.02). Interestingly, mortality was higher in CRT-P versus CRT-D patients with ischemic cardiomyopathy in all groups of patients. Until randomized data are available, this study might add a support value in selection of CRT-system. Moreover, this study illustrates that CRT-D therapy was associated with a significant lower all-cause mortality regardless of etiology and patient age [34].

## Atrial fibrillation and HF

Atrial fibrillation (AF) is a common and prognostically unfavorable comorbidity in patients with HF [35, 36]. Although restoration and maintenance of sinus rhythm would be ideal for these patients, several studies comparing rhythm and rate control have failed to show any advantage of rhythm control achieved with pharmacological therapy in terms of HF hospitalization or death [37, 38]. Catheter ablation is an established therapy for symptomatic AF patients who do not respond to medical therapy and have normal cardiac function [38, 39]. Furthermore, early rhythm-control therapy has been associated with a lower risk of adverse CV outcomes than usual care among patients with a history of CV disease within the first year of AF diagnosis [40]. Several recent studies have shown improvement in clinical outcomes after AF ablation in HF patients, highlighting the growing importance of the invasive approach in this patient population [39]. The recent Catheter Ablation vs. Standard Conventional Therapy in Patients with Left Ventricular Dysfunction (CASTLE-AF) trial was the first randomized trial investigating the impact of AF catheter ablation compared with medical therapy (rate or rhythm control) on mortality and hospitalizations in patients with HF. Symptomatic AF patients with (LVEF < 35%) participated in the study. A significant reduction in the composite endpoint of death and HF hospitalization was shown in the catheter ablation group compared with the medical therapy one [39]. More recently, the Catheter Ablation vs. Medical Rate Control in Atrial Fibrillation and Systolic Dysfunction (CAMERA-MRI) trial randomized patients with idiopathic cardiomyopathy and persistent AF who underwent CMR to either catheter ablation or rate control therapy [39]. There was a significant improvement in LVEF in patients who underwent catheter ablation compared with those who were randomized to drug therapy despite optimal rate control. Patients with no evidence of late gadolinium enhancement (LGE) on CMR had better improvements in their LV function after the ablation compared with those who had LGE. This result showed that CMR might be a strong tool to identify HF patients who may benefit more from catheter ablation [39]. However, the AMICA trial (Atrial Fibrillation Management in Congestive Heart Failure With Ablation) did not reveal any benefit of catheter ablation in patients with persistent AF and EF < 35. One year after ablation, LVEF displayed a comparable increase as in patients on best medical therapy without ablation [41]. Current guidelines recommend catheter ablation of AF to resolve LV dysfunction in AF patients when tachycardia-induced cardiomyopathy is highly likely, regardless of their symptom status (Class I recommendation) [38]. In conclusion, catheter ablation appears promising in patients with AF and HF. Some concerns remain regarding patient selection and standardization of the ablation procedure to best balance the risks and benefits of the procedure in this population.

## Has the maximum benefit been reached?

Patients with HF have a substantially shorter life expectancy than age-adjusted groups of patients without HF [42]. Intensive anti-neuroendocrine therapy has generally been successfully delivered in a stepwise fashion with the addition of the next agent in the recent years, which is the basis for the stepwise guideline recommendations [42]. An open question is whether intensive therapy with newer substances, such as SGLT2 inhibitors and angiotensin receptor/neprilysin inhibitors (ARNI), lead to a further reduction in HF outcomes or whether the beneficial effects have maximally reached and thus no further effect can be achieved by polypharmacotherapy. In a "meta-network analysis", which implies the comparison of all placebo-controlled trials with pooled placebo groups, the efficacy of individual substances was investigated [43]. This showed a 42% decrease in all-cause mortality with a fully complete combination of four HF agents [angiotensin-converting enzyme (ACE) inhibitors, beta-blocker, mineralocorticoid antagonist (MRA) with ivabradine] and 52% with ARNI added to beta-blocker and MRA therapy [43]. Furthermore, it has been shown that additional therapy adding an SGLT2 inhibitor and an ARNI leads to an extra benefit and increased life expectancy compared with conventional therapy with an ACE inhibitor, angiotensin receptor blocker (ARB) or beta-blocker, which is dependent on the patient's age at diagnosis of HF [44]. For example, it has been shown that when therapy is started at age 55, the increase in life expectancy is about 6–7 years [43, 44]. This clearly demonstrates that the use of different drugs mechanisms: anti-neuroendocrine therapy with beta-blockers and ACE inhibitors/ ARB, support of adaptive mechanisms with neprilysin inhibition or metabolic effects with SGLT2 inhibitors (partly unexplained) have achieved further additive benefits in addition to previous therapies [44]. Accordingly, drug “poly-pharmacotherapy” in CHF appears to be justified.

## Should we move to individualized rather than chronological treatment recommendation?

**Fig. 1:**
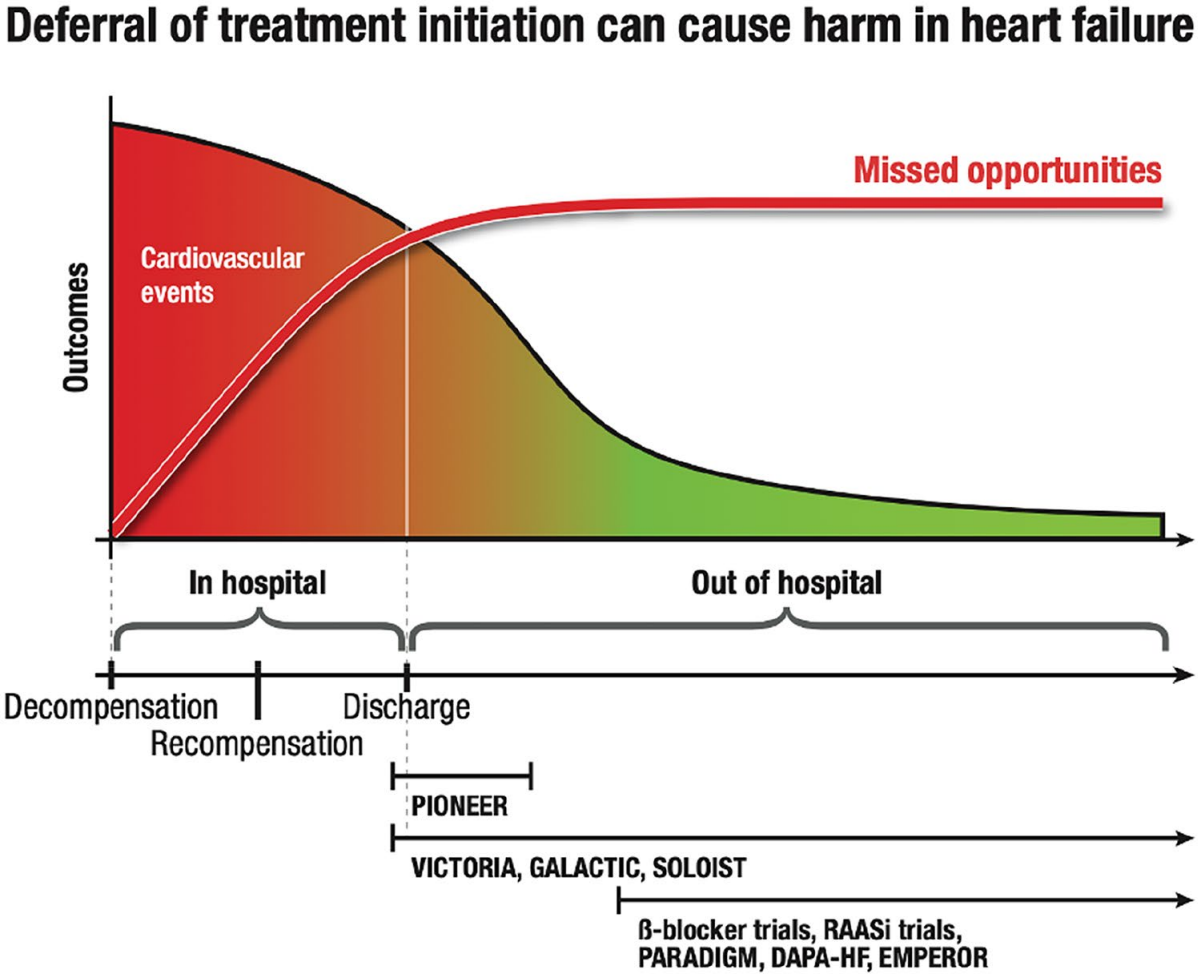
A schematic scheme of the association of outcomes, treatment initiation, missed opportunities and potential benefits of early intervention in HF patients. The Figure shows landmark HF trials during the period where patients were included in those studies. Data taken from [3, 4, 18, 21, 25, 57, 61]

**Fig. 2:**
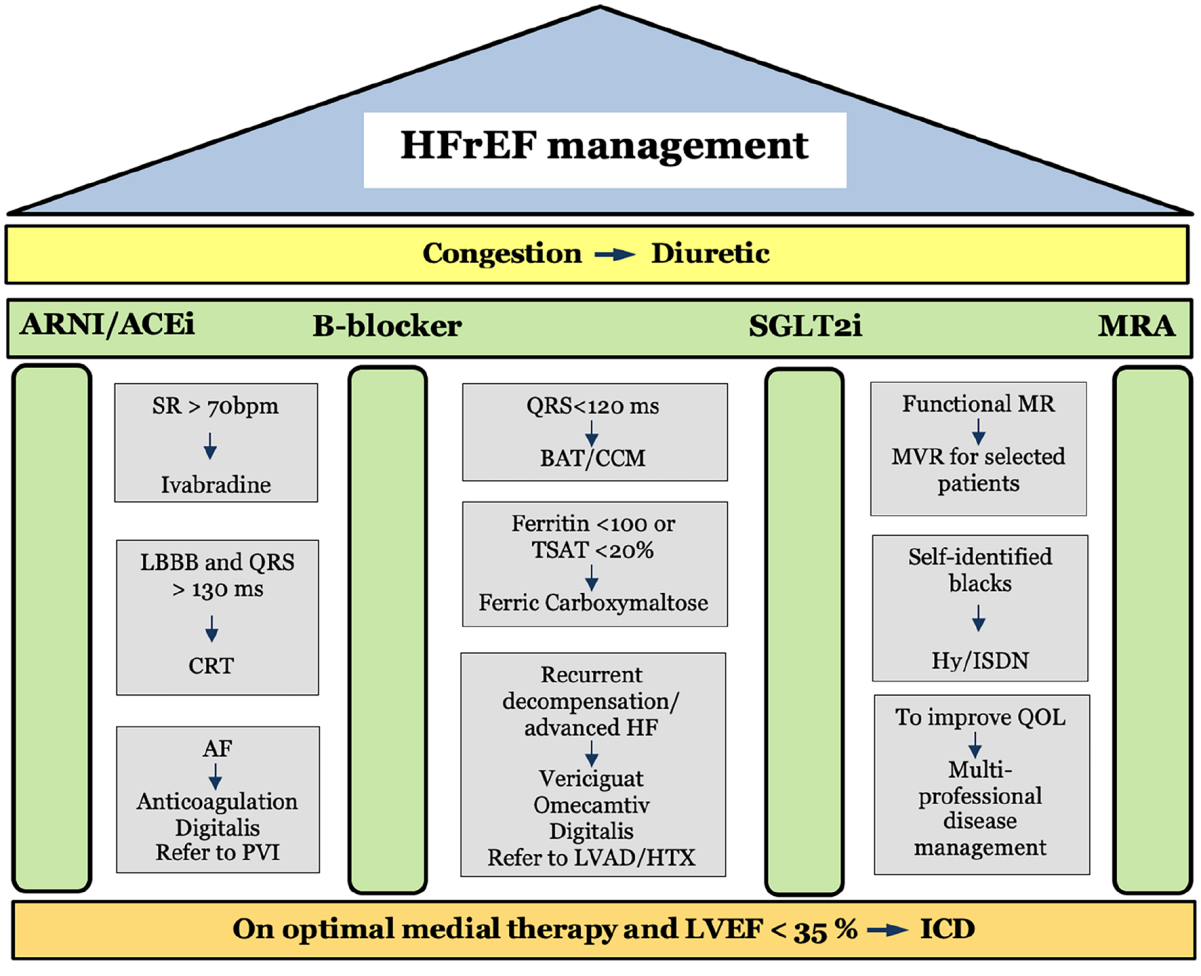
Overview on management of heart failure with reduced ejection fraction (HFrEF). ARNI/ACEi/ARBs, B-blockers, MRA, and SGLT2 inhibitors are indicated as soon as possible for all HFrEF patients (4 pillars of optimal HFrEF medical therapy). This is followed by devices or other drug therapies. Subgroups with: SR and HF > 70, LBBB with QRS > 130 ms, recurrent decompensation/advanced HF, self-identified blacks will treat accordingly. Comorbidities such as iron deficiency, MR, AF will treat appropriately. Diuretic is given for severe congestion across the whole spectrum. An ICD should be implanted due to the risk of malignant arrhythmias if left ventricular function is consistently reduced (LVEF < 35%) *ACE-I*: angiotensin-converting enzyme inhibitor, *AF*: atrial fibrillation, *ARNI*: angiotensin receptor/neprilysin inhibitor, *BAT*: Baroreflex activation therapy, *B-blocker*: beta-blocker, *CCM*: Cardiac contractility modulation, *CRT*: cardiac resynchronization therapy, *HF*: heart failure, *HTX*: heart transplantation, *Hy* hydralazine, *ICD*: implantable cardiac defibrillator, *ISDN*: Isosorbide dinitrate, *LBBB*: left bundle branch block, *LVAD*: left ventricular assist device, *LVEF*: left ventricular ejection fraction, *MR*: mitral regurgitation, *MRA*: mineralocorticoid receptor antagonist, *MVR*: mitral valve repair, *PVI*: pulmonary vein isolation, *QoL*: quality of life, *SGLT2*: sodium–glucose co-transporter 2, *SR*: sinus rhythm, *TSAT*: transferrin saturation

Previous guideline recommendations were based on the time when the evidence for effective therapies was generated. For example, in early trials of ACE inhibitors, such as CONSENSUS-I, therapy was compared only with diuretics and occasionally digitalis therapy [45]. In this respect, in the previous HF guidelines ACE inhibitor was always in the first place. Recently, the CIBIS III study [46] showed a similar morbidity and mortality statistics with concomitant initiation of enalapril or bisoprolol. Therefore, current guidelines recommend beta-blockers and ACE inhibitors as an initial therapy [42]. The initiation of therapy in stable patients, long after decompensation, often leads to delayed start of drug therapy and patients are not well treated especially after discharge, where a particularly high mortality and hospitalization rate occurs [47, 48]. This due to the evidence from the most controlled clinical trials, which was generated only after discharge in stable ambulatory patients long time after their hospitalization with worsening HF (Fig. 1). Due to registries showing benefits, when treatment is started immediately after decompensation and even before discharge, an early initiation of therapy could be recommended. Most importantly, novel treatments with SGLT2 inhibitors and ARNI, even at low starting doses, resulted in a statistically significant end-point reduction within the first 30 days after randomization [49, 50]. This highlights that subsequently therapy with all four foundational HFrEF drugs, namely ARNI, beta-blockers, MRA, and SGLT2 inhibitors, should therefore be achieved within 4 weeks in HFrEF patients [51, 52]. Other HF medications should also be implemented promptly according to patient characteristics to improve outcomes [51, 52]. This approach was already implemented in the new Canadian Cardiovascular Society HF guidelines update, which strongly recommended that HFrEF patients should be initially treated with combination therapy from each of the following categories (ARNI/ACEI, B-blocker, MRA and SGLT2 inhibitor) [53]. Other therapies should be individualized to subgroups based on clinical scenario (ICD/CRT, ivabradine in sinus rhythm above 70 beats/min, assist systems, heart transplantation, etc.). New substances, such as vericiguat and omecamtiv mecarbil, have achieved statistically significant effects but with a limited effect size in reducing the primary endpoint but neutral results in key secondary outcomes, such as CV and all-cause death. Presently, it is open whether omecamtiv will get a market approved therapeutic agent. It would be conceivable that vericiguat and potentially also omecamtiv could be recommended for patients with advanced HFrEF after a recent hospitalization for worsening of heart failure. A summary of all possible interventions including those for subgroups and those for special situations is shown in Fig. 2.

## Heart failure is an urgency needing rapid intervention

Despite remarkable improvements in HF care, it is still a globally progressive condition with more than 37 million patients worldwide [42, 54]. According to the clinical presentation, the HF syndrome can be classified into acute or chronic [54], and further into acute HF (AHF) defined as new (de novo) HF or worsening of symptoms and signs of pre-existing chronic HF (CHF) [42]. Worsening of CHF accounts for 80–90% of those patients hospitalized while only 10–20% have new-onset or advanced HF [54, 55]. Currently, hospitalization due to AHF is still associated with poor outcomes, with re-hospitalization rates and 1-year mortality up to 30% [54–56]. Accordingly, the importance of early diagnosis and rapid intervention has been highlighted in the recent HF guidelines; however, timelines were not stated [42, 57]. A recent multinational registry showed that regardless of admission via the emergency department, normal ward or the intensive care unit in Western Europe, the median time to initiation of a diuretic was around 3 h [56]. “Door to Diuretic” (D2D) time was significantly longer in North America compared with other regions like Eastern Europe and Southeast Asia [56]. A Japanese registry showed that rapid volume unloading within the first 30 min is associated with improved prognosis than delaying the first relieving diuretic therapy beyond one hour [58]. The authors recommend that it is useful to work with strict timelines in acute decompensation and to complete patient evaluation and diagnostics within 5 min, and subsequently to start decongestion therapy within the first 20 min [58]. These data strongly support the previously mentioned recommendation of rapid initiation of therapy in patients with AHF, which can essentially be achieved by rapid diuretic therapy [57, 59]. An early decrease of wall tension, and consequently neuroendocrine activation, with a potential reduction of myocyte damage could provide long term benefit [59, 60], in addition to the fact that rapid relief of symptoms of congestion is an ethical requirement. Therefore, the concept of "time is muscle" also seems to be of importance in AHF, as is well established in acute coronary syndromes (58). Consequently, there is a clinical challenge and an urgent need for early identification of HF patients and rapid interventions to interrupt the progression of this disease.
